# Emersion Induces Nitrogen Release and Alteration of Nitrogen Metabolism in the Intertidal Genus Porphyra

**DOI:** 10.1371/journal.pone.0069961

**Published:** 2013-07-26

**Authors:** Jang K. Kim, George P. Kraemer, Charles Yarish

**Affiliations:** 1 Departments of Ecology and Evolutionary Biology and Marine Sciences, University of Connecticut, Stamford, Connecticut, United States of America; 2 Department of Environmental Studies, Purchase College, Purchase, New York, United States of America; Algenol Biofuels, United States of America

## Abstract

We investigated emersion-induced nitrogen (N) release from *Porphyra umbilicalis* Kütz. Thallus N concentration decreased during 4 h of emersion. Tissue N and soluble protein contents of *P. umbilicalis* were positively correlated and decreased during emersion. Growth of *P. umbilicalis* did not simply dilute the pre-emersion tissue N concentration. Rather, N was lost from tissues during emersion. We hypothesize that emersion-induced N release occurs when proteins are catabolized. While the δ^15^N value of tissues exposed to emersion was higher than that of continuously submerged tissues, further discrimination of stable N isotopes did not occur during the 4 h emersion. We conclude that N release from *Porphyra* during emersion did not result from bacterial denitrification, but possibly as a consequence of photorespiration. The release of N by *P. umbilicalis* into the environment during emersion suggests a novel role of intertidal seaweeds in the global N cycle. Emersion also altered the physiological function (nitrate uptake, nitrate reductase and glutamine synthetase activity, growth rate) of *P. umbilicalis* and the co-occurring upper intertidal species *P. linearis* Grev., though in a seasonally influenced manner. Individuals of the year round perennial species *P. umbilicalis* were more tolerant of emersion than ephemeral, cold temperate *P. linearis* in early winter. However, the mid-winter populations of both *P. linearis* and *P. umbilicalis,* had similar temporal physiological patterns during emersion.

## Introduction

Marine algae release O_2_ and CO_2_ into the atmosphere via photosynthesis and biological respiration, respectively. These, however, are not the only materials released. For example, seaweeds emit iodine, generating aerosols that may affect climate [1]. Our earlier findings suggested another contribution from intertidal seaweeds to the environment [2]. During emersion, the tissue nitrogen (N) content of *Porphyra umbilicalis* Kütz decreased. If *P. umbilicalis* had simply used internal N for growth during emersion (when there was no N uptake), the drop in tissue N concentration would represent dilution of the tissue N concentration. However, our mass balance calculations indicated that measured declines in tissue N concentration of *P. umbilicalis* would require a growth rate of 50–160% d^−1^ [2]. Since maximum measured growth rates were only 2.4% d^−1^, we are confident that dilution of stored N cannot explain the reduction of tissue N, and that P. umbilicalis lost N during emersion. This is the first study showing a possibility of N release into the atmosphere by an intertidal seaweed.

Nitrogen might be lost in several forms during emersion. Leakage of internal pools of inorganic N nitrate (NO_3_^−^) and nitrite (NO_2_^−^) is one possibility, though these are present at very small pool sizes (0.1 µmol g^−1^ FW) [3], arguing that this form alone cannot explain the much greater total observed loss [2]. Ammonium (NH_4_^+^) is, likewise, present at very low concentrations (e.g., 1 µmol g^−1^ FW) [4]. Unlike NO_3_^−^ and NO_2_^−^, however, ammonium may be volatilized as NH_3_, especially if photosynthesis reduces internal pH [5]. An organic form of N could also be lost during emersion. Amino acid concentrations are known to decline during emersion [3], though amino acids are present internally at only one to two orders of magnitude greater concentration than dissolved inorganic pools. Since the original report of N loss [2] analyzed samples at the end of the period of emersion and before resubmergence, all forms of unvolatilized N (i.e., surface-associated NO_3_^−^, NO_2_^−^, organic N-containing compounds) were captured by the tissue N analysis and can, therefore, be ruled out as the vectors for N loss.

Finally, N might be lost via denitrification conducted by a bacterial biofilm. Denitrification reduces NO_3_^−^ to N_2_ gas under anaerobic conditions [6], though this may also occur under aerobic conditions [7], and has been conducted by periphyton on fresh water and marine macrophytes [8]–[11]. Denitrifying bacteria could act on either directly released NO_3_^−^ or N initially released by the deamination of N-containing organic compounds. The naturally occurring stable isotopes ^15^N and ^14^N have helped elucidate N metabolic pathways and processes in both laboratory and field situations [12], [13]. Denitrifying bacteria preferentially reduce nitrate containing the lighter isotope (^14^NO_3_^−^) over the heavier one (^15^NO_3_^−^) [14]. If the N lost during emersion by *Porphyra umbilicalis* [2] is released into the atmosphere via bacterial denitrification,^ 14^N should disappear from the cells more rapidly than ^15^N. Therefore, the nitrogen isotopic fractionation in the *Porphyra* tissue should increase during emersion if N exits the thallus as a result of denitrification.

The uptake of inorganic nitrogen is central to the growth and reproduction (i.e., the fitness) of marine macroalgae, and to the resupply of N lost during emersion of *Porphyra*. Once NO_3_^−^ is taken into the algal thallus, nitrate reductase (NR) represents the first enzymatic step in the assimilation of N into organic form. The final assimilatory step is mediated by glutamine synthetase (GS). In addition, GS captures NH_3_ generated via photorespiration [15]. Measurement of NO_3_^−^ uptake, and NR and GS activities shed light on both the impact of emersion on N metabolism, and on the recovery following resubmergence.

Intertidal seaweeds may employ different strategies to survive the stresses of emersion. Unlike higher plants, where leaves close their stomata to conserve water, seaweeds are subject to significant evaporative water loss (sometimes exceeding 80%) during emersion at low tide [2], [16]–[20]. The thickness of the algal thallus can be a morphological strategy to reduce emersion stress [20], [21]. Two thalli differing in thickness should have similar total rates of water loss (g H_2_O min^−1^) under the same physical conditions. However, the relative rate of water loss (g H_2_O g^−1^ tissue min^−1^) of the thinner thallus will be greater, and the thinner will experience earlier and ultimately greater emersion stress.

Intertidal species such as *Porphyra* species may coexist in time (same season) and/or space (same vertical elevation in the intertidal zone). *Porphyra umbilicalis* occurs throughout the year in diverse coastal and estuarine habitats, *i.e*. from the lower to upper intertidal zones in the Gulf of Maine and the upper intertidal in Long Island Sound [22]. The thallus cross sectional thickness of this species is 80–110 µm [23]–[26]. *Porphyra linearis* forms ephemeral populations of gametophytes during the winter months within the upper intertidal zones of open coastal habitats in New England [22], and is much thinner (25–50 µm).

Within the general goal of elucidating the N metabolism of intertidal macroalgae and the flux of N through this ecologically important assemblage, this study addressed the following specific, inter-related objectives: (a) validate prior measurements suggesting N loss from thalli of *P. umbilicalis* during emersion [2]; (b) determine whether N loss from thalli occurs via denitrification; (c) evaluate the degree to which emersion alters the uptake of nitrate (NO_3_^−^) and the activity of N assimilation enzymes in *P. umbilicalis* and the co-occurring intertidal species *P. linearis*.

## Materials and Methods

### Algal Materials and Culture Condition

Both *P. linearis* and *P. umbilicalis* were collected from the same rocky habitat in the upper intertidal zone at Rye, New Hampshire (43° 00′43.3″N, 70° 44′04.6″W; an open public access area requiring no permission to collect seaweed), in November and December, 2007 for NR experiments, and in January and February, 2008 for GS experiments. Our field studies did not involve endangered or protected species. Individual thalli were removed singly from rocks haphazardly chosen within the intertidal zone. For the N release experiment, another collection of *P. umbilicalis* was made from the same rocky habitat in February 2008. Thalli were transported to the laboratory on ice in a cooler, and there cleaned of epiphytes by rinsing with running seawater and gently rubbing with cotton balls.

Experiments were conducted in a greenhouse at the University of Connecticut at Avery Point, Groton, Connecticut. Samples were either kept continuously submerged or exposed for 4 h periods on a semi-diurnal emersion cycle using a tide simulating apparatus [26]. The tide simulating apparatus and the identical apparatus but providing constant submergence sat in a large outer tank connected to a chiller to maintain constant 10°C temperature. The culture medium was 0.45 µm-filtered seawater with von Stosch’s enrichment (VSE) [27] without N or phosphorus (P). Concentrations of dissolved inorganic N and P in the ambient seawater collected from Avery Point were analyzed and were below detection limits during the experiments. Nitrogen and P levels were regulated by the addition of KNO_3_ and Na_2_HPO_4_ to 30 and 3 µM, respectively, to ensure a stable supply and non-limiting nutrient status in the algal tissues. Experiments used the optimal temperature for the growth of both species (10°C) [28], [29]. The maximum light intensity measured in the greenhouse by a Li-Cor LI-1000 (Li-Cor, Inc., Lincoln, Nebraska, U.S.A.) photometer was 1220 µmol photons m^−2^s^−1^ during this winter period. The air temperature and humidity during exposure were 18±3°C and 6–30%, respectively. Stocking density for each treatment was 0.5 g L^−1^.

### Acclimation

*Porphyra* tissues were acclimated to the experimental conditions (simulated tidal cycle including emersion, or constant submergence) for 5–7 d. Filtered seawater containing VSE with 30 µM of nitrate and 3 µM of phosphate was replaced daily to ensure a stable nutrient status in the algal tissues during acclimation. Thalli were randomly assigned to either a constant submersion or an emersion treatment. Those thalli assigned to the emersion treatment were exposed to air for 4 h twice daily.

### N Release Experiments

Experiments were begun at 0700 h. To validate prior measurements suggesting N loss during emersion, replicate acclimated thalli of *P. umbilicalis* were exposed to continuous submergence or 4 h periods of emersion. Samples under the emersion treatment were exposed to air from 1000 to 1400 h, and 2200 to 0200 h (final water loss of approximately 90±5%), while controls remained submerged during the 27 h experiment.

Each tide simulating apparatus contained 18 independent compartments (three rows of six compartments), each containing ca. 2.5 liters of seawater. During the experiments, the culture medium (VSE with 30 µM nitrate and 3 µM phosphate), was changed at 0700, 1000, 1400 and 1730 h to ensure sufficient nutrients in the culture media. Our growth rate calculations, coupled with measured tissue N concentrations, indicate that nitrogen concentrations in the incubation medium remained over 90% of initial concentration throughout the experiment.

Tissue samples were taken at the outset of the experiment (initial sample), at sunrise (0700 h), immediately before exposure (1000 h), at the end of the exposure period (1400 h) and the following morning (1000 h). Thalli from six randomly selected compartments were harvested for tissue analyses at each sample time (true replication). Growth rate was determined as biomass increase after blotting thalli dry with paper towels. For analysis of total tissue N content, samples were dried at 55°C before being ground. N content of powdered thallus samples was measured using Perkin Elmer 2400 series II CHNS/O elemental analyzer, with acetanilide as standards. For analysis of total tissue protein content, approximately 0.25 g FW of tissue samples was ground with 1 mL of protein extraction buffer (50 mM Tris-HCl pH 8.0, 150 mM NaCl, 10 mM β-mercaptoethanol, 1 mM magnesium acetate and 1 mM imidazole). A 0.1 mL aliquot of the protein extract was combined with 5 mL of protein reagent (0.01% (w/v) Coomassie Brilliant Blue G-250, 4.7% (w/v) ethanol, and 8.5% (w/v) phosphoric acid). The protein contents were determined by measuring absorbance at 595 nm [30]. Known concentrations of bovine serum albumen were used to construct a standard curve.

To determine whether N loss from thalli occured via denitrification, the δ^15^N values of dried, powdered samples were analyzed by the University of California at Davis Stable Isotope Facility (Davis, CA, U.S.A.). The standard metric by which differences in N isotope concentrations are presented is δ^15^N (‰):
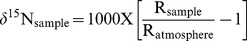
where R is the ratio of ^15^N to ^14^N (‰). Atmospheric N_2_ consists of N in an ^15^N/^14^N ratio of 3.67×10^−3^.

### NR and GS Experiments

The degree to which emersion altered NO_3_^−^ uptake and the activity of the N assimilatory enzymes NR and GS in *P. umbilicalis* and the co-occurring intertidal species *P. linearis* were investigated using the same experimental apparatus and set-up were used (see ***N release experiments***, above) except for the shortened experiment duration (0700–21∶30) and fewer replicates (*n = *3). Tissue and water samples were collected at sunrise (0700 h), immediately before exposure (1000 h), at the end of exposure (1400 h) and 0.5, 1.5, 3.5 and 7.5 h after re-submergence. Water and tissue samples were collected simultaneously from the same compartments. At each time point, all three compartments were completely removed (true replication). Water samples from the incubation medium were analyzed for inorganic nitrate by using a SmartChem Discrete Analyzer (Westco Scientific Instruments, Inc., Brookfield, CT, U.S.A.).

*In vivo* NR activity was measured in this study [31]–[33]. Tissue samples (0.5 g) were incubated at room temperature in 22 mL of incubation medium in a dark flask (0.06 M KNO_3_, 0.1 M KH_2_PO_4_ and 0.5% 1-propanol (v:v), pH 7.0). To insure all the tissues were completely bathed by incubation medium, the algal tissues were cut into smaller pieces (<1 cm^2^). The medium was gently mixed every 5 min. The medium was briefly and gently flushed with N gas to purge oxygen, and the top was sealed with Parafilm®. One mL was removed from each replicate at half-hour intervals, and one mL of stop buffer was added (0.5 mL of 0.1% (w:v) napthyl ethylene diamine in 1 N HCl, 0.5 mL of 5% (w:v) sulfanilamide in 1 N HCl). The flasks were reflushed with N gas and resealed with Parafilm® after sampling. To quantify the conversion of nitrate to nitrite, absorbance at 540 nm was measured with a Spectronic Genesys 5 spectrophotometer (Spectronic Instruments, Rochester, NY, U.S.A.). Absorbance readings were calibrated against a nitrite standard curve.

The GS activity was measured by the *in vitro* assay [34], [35]. Samples of ca. 0.2 g FW tissue were ground in 2 mL of ice-cold extraction buffer (50 mM imidazole, pH 7.3, 0.14% [v/v] 2-mercaptoethanol, 10 mM MnCI2, 10% [v/v] glycerol, 0.03% [v/v] Tween-20, 1% [w/v] PWP). Homogenates were centrifuged to clear cell debris at 2,500g for 30 min at 4°C. An aliquot of the resulting tissue extract was added to the reaction cocktail (final concentrations: 470 mM imidazole, pH 7.3, 26 mM glutamine, 3 mM MnCI2, 0.4 mM ADP, 20 mM arsenate, 26 mM hydroxylamine) and incubated at 35°C. Aliquots were removed from the reaction mixture at 10–20 min intervals, added to an equal volume of stop reagent (2 N HCI, 5% [w/v] trichloroacetic acid, 13.3% [w/v] FeCI2) and quantified by spectrophotometry at 540 nm and compared to fresh solutions of gamma-glutamyl hydroxamate. In addition to measurements of enzyme activity, thallus growth rate and nitrate uptake were also recorded during both the NR and GS experiments.

### Statistical Analysis

ANOVA was used to evaluate the influence of time and emersion. Both time and emersion treatment were fixed factors, with time re-coded as a categorical independent variable. A repeated measures ANOVA was not used because the randomly selected compartments from each sample were true replicates (i.e., thalli were not resampled). The effects of the independent variables (time during the experiment and emersion treatment) were evaluated via measurements of growth rate, nitrate uptake rate, NR and GS activity, tissue δ^15^N and N content, and protein contents. The two *Porphyra* species were not compared statistically since the experiments were performed separately in time (one-two weeks) for each species. Our results (below) suggest that time of year may influence the physiology of *Porphyra*. *Post hoc* analysis via Tukey’s HSD test was used to make pairwise comparisons of treatment means when ANOVA indicated a significant effect of either independent variable. Prior to ANOVA, data were examined for homogeneity of variance. The data sets differed in relative variability, with nitrate uptake and NR activity generally requiring ln-transformation toward meeting the requirement of homogeneity of variance terms. In several cases, transformation (ln, square root both applied) did not produce homogenous variances. However, ANOVA procedures are robust with regard to this violation when the sample sizes are equal, as ours were [36]. Regression was used to determine the degree to which tissue N and protein were linked, as well as the connection between nitrate uptake rate and NR activity and GS activity. Grubb's test was used to exclude outlying data points (a maximum of one outlier per graph). ANCOVA was employed to test for the homogeneity of slopes of the control and emersion treatments for each *Porphyra* species. All statistical analyses were conducted using Statistica 5.1 (StatSoft, Tulsa, OK, U.S.A.).

## Results

### N Release Experiments

**Figure 1:**
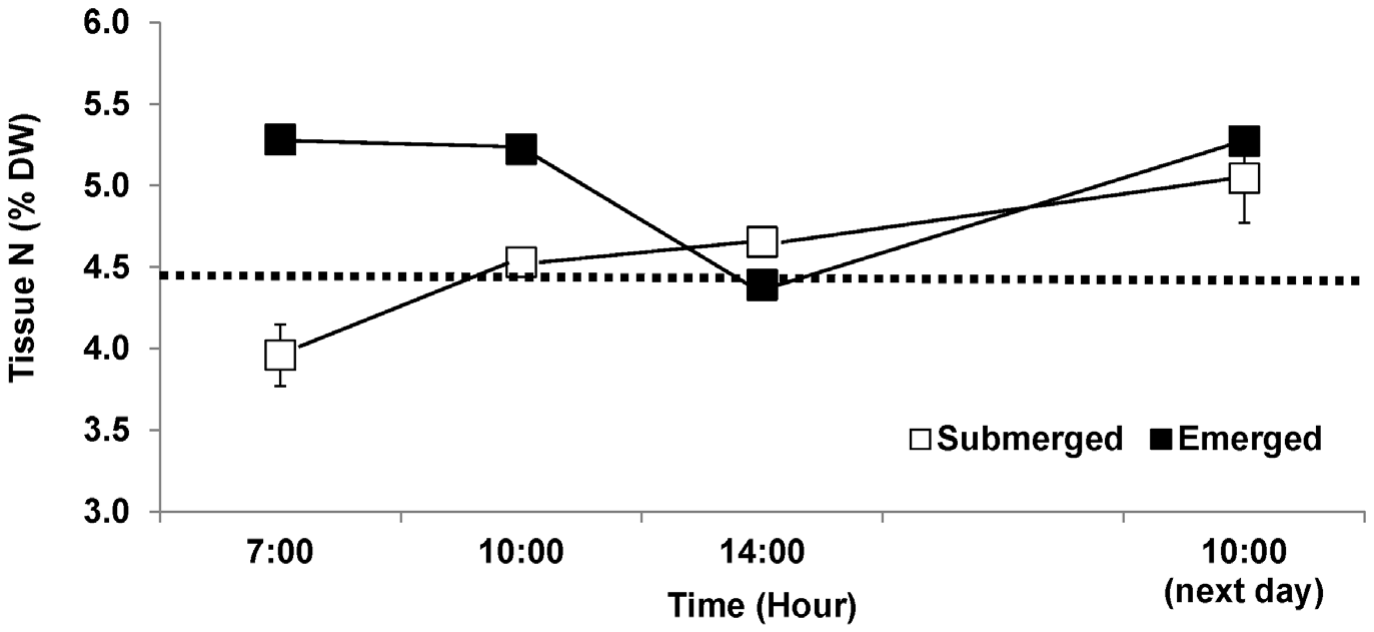
Tissue nitrogen protein content in *Porphyra umbilicalis* tissues from the upper intertidal zone and different emersion treatments. *Porphyra* was cultivated under ambient sunlight in a greenhouse, with sunrise and sunset at ca. 07∶00 and 18∶00, respectively. Filled squares represent the emersion treatment in which thalli were exposed to air on a semi-diurnal cycle (10∶00–14∶00, 90±5% water loss). Open squares (controls) remained submerged (0% water loss). Dotted line indicates the initial tissue N content (before the acclimation began). Error bars represent ± one standard deviation.

Tissue N contents were influenced by time, emersion and by the interaction of these two factors (Table 1). The effect of emersion was evident even before the outset of the experiment; tissue from the control treatment was 11% lower (p = 0.017) than the pre-acclimation measurements (Fig. 1), while tissue from the emersion treatment was 18% higher (ANOVA of tissue N content as a function of source, F_1,11_ = 43.2, p<<0.0001, post hoc comparison of control and emersion treatments, p = 0.0009).

**Table 1:** Results of analysis of variance examining the effects of emersion and time on δ^15^N, tissue nitrogen and soluble proteins of *Porphyra umbilicalis* from the upper intertidal zones.

Variable	Factor	F	*p*-value
Tissue N	Tissue source atexperiment start^1^	43.2	**<<0.0001**
Tissue N	Emersion^2^	57.59	**<0.001**
	Time of Day	20.55	**<0.001**
	E X T	24.72	**<0.001**
Soluble protein	Emersion	35.99	**<0.001**
	Time of Day	8.39	**<0.001**
	E X T	6.00	**0.002**
δ^15^N	Emersion	15.37	**<0.001**
	Time of Day	1.10	0.346
	E X T	1.43	0.250

1 Tissue source refers to pre-acclimation, control-acclimated, and emerion-acclimated tissues.

2 Control vs. 90% water loss.

Significant differences are shown in bold with p values.

**Figure 2:**
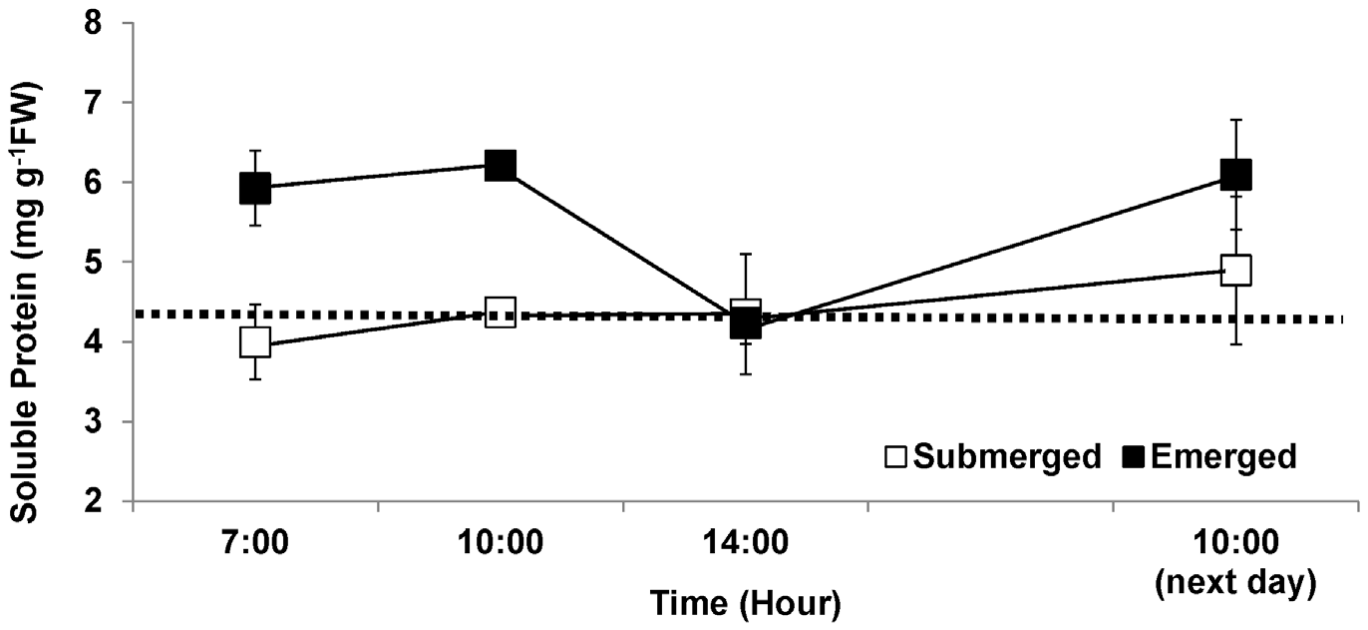
Soluble protein content in *Porphyra umbilicalis* tissues from the upper intertidal zone and different emersion treatments. *Porphyra* was cultivated under ambient sunlight in a greenhouse, with sunrise and sunset at ca. 07∶00 and 18∶00, respectively. Filled squares represent the emersion treatment in which thalli were exposed to air on a semi-diurnal cycle (10∶00–14∶00, 90±5% water loss). Open squares (controls) remained submerged (0% water loss). Dotted line indicates the initial tissue N content (before the acclimation began). Error bars represent ± one standard deviation.

**Figure 3:**
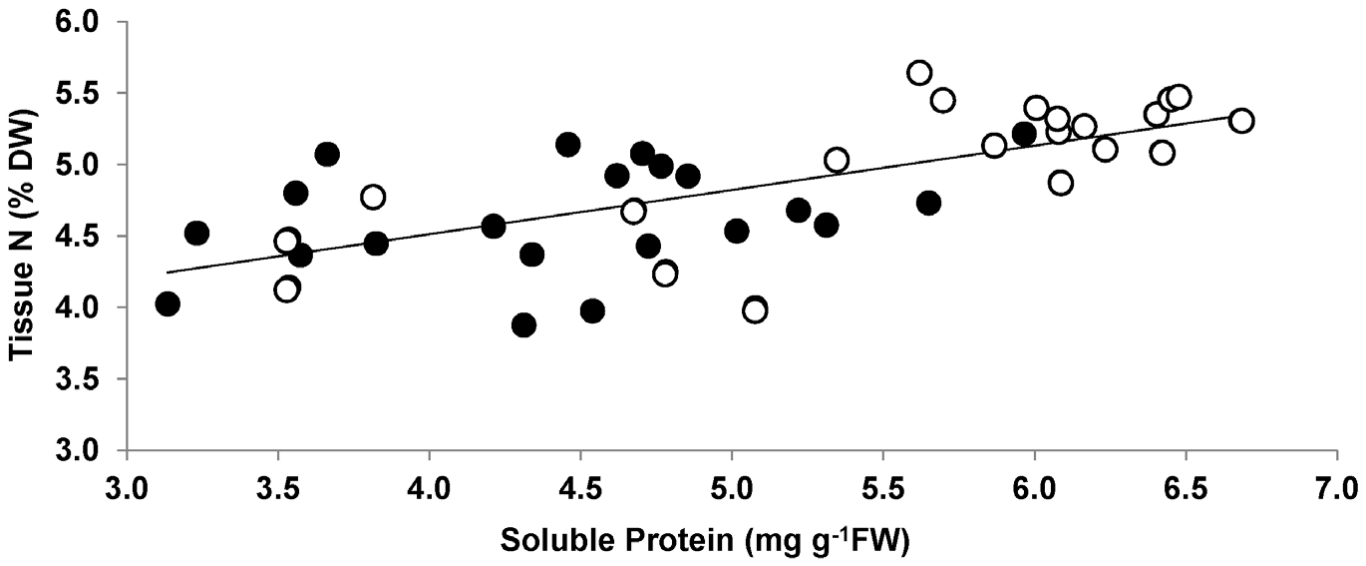
Soluble protein vs. tissue N of *Porphyra umbilicalis* from the upper intertidal zone. All samples from the experiment period are pooled here. *Porphyra* was cultivated under ambient sunlight in a greenhouse, with sunrise and sunset at ca. 07∶00 and 18∶00, respectively. Filled circle: control, open circle: emerged. Regression is highly significant (F_1,40_ = 35.3, p<<0.001).

Four hours of emersion during the experiment (1000–1400 h) caused a significant reduction in tissue N content. The value of tissue N at the end of the 4 h emersion period averaged only 84% of the pooled values of the other emersion treatment samples. When values at the end of the emersion period were excluded (to eliminate the short-term, interactive effects of time and emersion stress on tissue N), tissues from the emersion treatment possessed 11% more N than did the controls (pooled across treatment; Fig. 1). Soluble protein content was also significantly influenced by time, emersion and combination of these two factors (Table 1). Similar to tissue N, time and emersion treatment interacted significantly, a result of the significant drop in soluble protein contents across the emergent period. The tissue protein value at the end of the emersion period was on average only 69% of the pooled values of other emersion treatment samples. After removal of the tissue protein data obtained at the end of emersion period, protein contents of tissues from the control (submerged) treatment were significantly lower than those of emerged treatment (Table 1); the protein content of the control averaged 74% of the emersion treatment (Fig. 2). Soluble protein content was positively correlated with total tissue N (F_1,40_ = 35.3, p<<0.0001, R^2^ = 0.47; Fig. 3).

**Figure 4:**
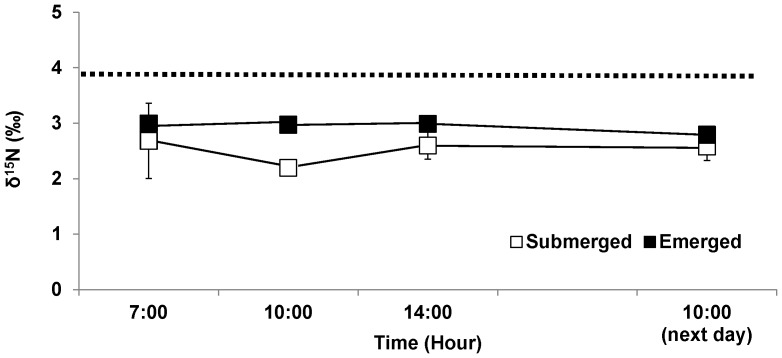
δ^15^N in *Porphyra umbilicalis* tissues from the upper intertidal zone and different emersion treatments. *Porphyra* was cultivated under ambient sunlight in a greenhouse, with sunrise and sunset at ca. 07∶00 and 18∶00, respectively. Filled squares represent the emersion treatment in which thalli were exposed to air on a semi-diurnal cycle (10∶00–14∶00, 90±5% water loss). Open squares (controls) remained submerged (0% water loss). Dotted line indicates the initial tissue N content (before the acclimation began). Error bars represent ± one standard deviation.

The δ^15^N value of tissues exposed to emersion treatment was significantly higher than that of the continuously submerged control (p<0.001), but δ^15^N was not affected by time (p>0.05). The δ^15^N in the tissues from the emersion treatment averaged 2.94‰ as compared to the control 2.51‰, a difference of 17% (Fig. 4; Table 1). The difference between field-collected tissues (δ^15^Nvalue = 3.825±0.667) assayed immediately and the starting values for the control (δ^15^Nvalue = 2.982±0.244) and the emersion treatment (δ^15^Nvalue = 2.996±0.339) reflect the difference in the nitrogen source (dissolved inorganic nitrogen in nearshore Rye, NH water vs. Sigma-Aldrich Co. KNO_3_ used in laboratory culture).

### NR Experiments

#### Growth

**Figure 5:**
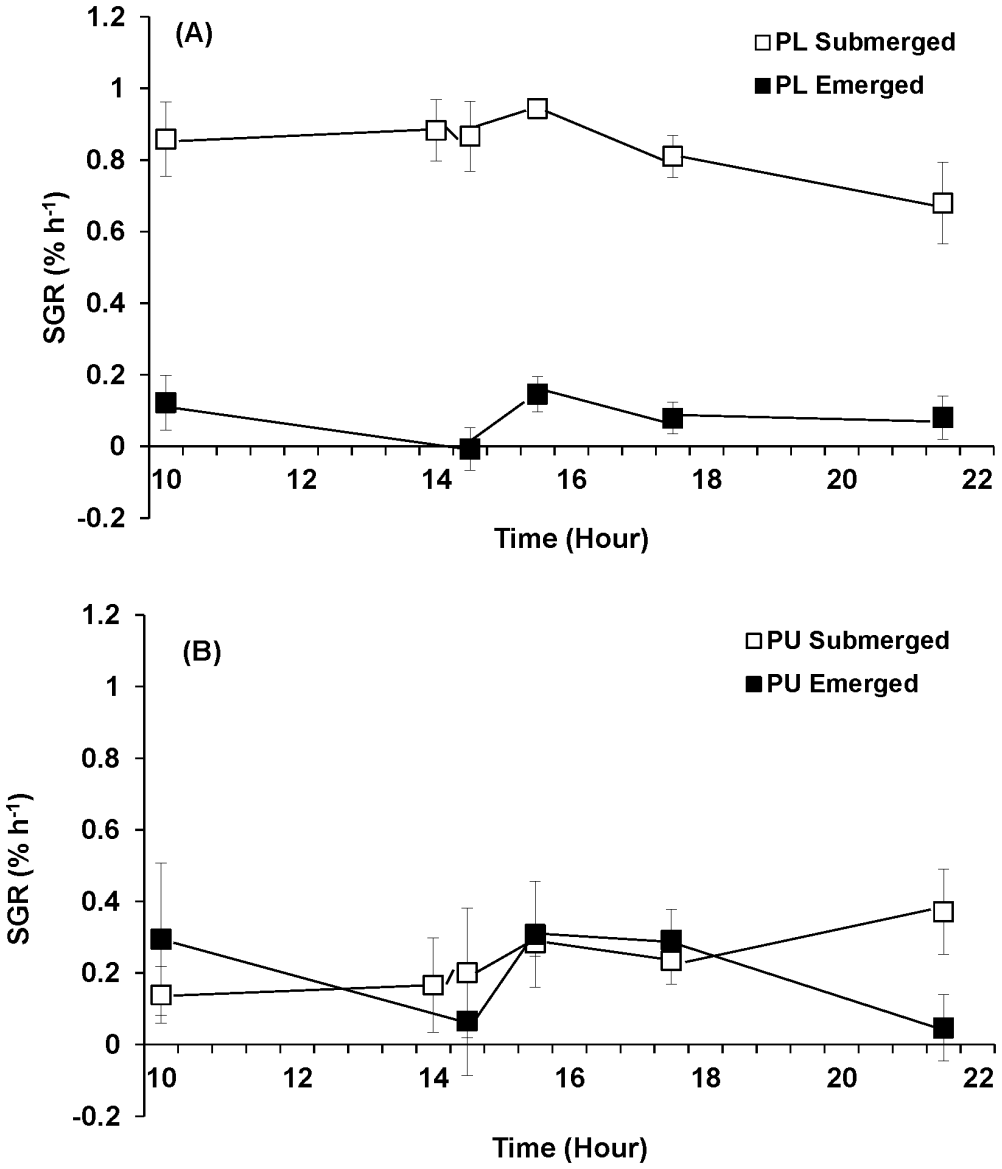
Growth rates of *Porphyra linearis* (PL) (A) and *P. umbilicalis* (PU) (B) from the upper intertidal zone during nitrate reductase experiments using thalli collected from the early winter (Nov. -Dec.) population. *Porphyra* was cultivated under ambient sunlight in a greenhouse, with sunrise and sunset at ca. 07∶00 and 18∶00, respectively. Filled squares represent an emersion treatment which was exposed to air at a semi-diurnal cycle (10∶00–14∶00). All controls, open squares, remained submerged. Error bars represent ± one standard deviation.

Growth rate at each sampling was calculated against the initial weight at 0700 h. Emersion significantly affected the growth of *Porphyra linearis* (Table 2). When continuously submerged, *P. linearis* grew 0.84% h^−1^ while *P. umbilicalis* grew on average 43% slower (0.48% h^−1^). However, when *P. linearis* experienced 90% water loss during emersion, the growth rate dropped to 0.08% h^−1^, only 10% of control rates, while *P. umbilicalis* still grew 0.27% h^−1^, approximately 55% of control rates (Fig. 5).

**Table 2:** Results of analysis of variance examining the effects of emersion and time on nitrate uptake, NR and GS activities of *Porphyra linearis and P. umbilicalis* from the upper intertidal zones.

Variable		Factor	F	p-value
Growth from	*P. linearis*	Emersion^3^	748.9	**<0.001**
NR Experiment		Time of Day	4.60	**0.009**
		E X T	1.98	0.138
	*P. umbilicalis*	Emersion	0.98	0.332
		Time of Day	1.52	0.234
		E X T	3.70	**0.019**
Nitrate Uptake	*P. linearis*	Emersion	87.60	**<0.001**
from NR		Time of Day	25.35	**<0.001**
Experiment		E X T	0.86	0.505
	*P. umbilicalis*	Emersion	34.69	**<0.001**
		Time of Day	238.65	**<0.001**
		E X T	11.24	**<0.001**
NR activity	*P. linearis*	Emersion	58.51	**<0.001**
		Time of Day	8.90	**<0.001**
		E X T	5.07	**0.001**
	*P. umbilicalis*	Emersion	12.12	**0.002**
		Time of Day	12.03	**<0.001**
		E X T	3.99	**0.015**
Growth from	*P. linearis*	Emersion	15.14	**0.001**
GS Experiment		Time of Day	6.81	**0.001**
		E X T	4.57	**0.008**
	*P. umbilicalis*	Emersion	34.84	**<0.001**
		Time of Day	3.26	**0.024**
		E X T	2.32	0.089
Nitrate Uptake	*P. linearis*	Emersion	17.05	**<0.001**
from GS		Time of Day	13.72	**<0.001**
Experiment		E X T	9.30	**<0.001**
	*P. umbilicalis*	Emersion	57.73	**<0.001**
		Time of Day	10.89	**<0.001**
		E X T	4.68	**0.008**
GS Activity	*P. linearis*	Emersion	23.30	**<0.001**
		Time of Day	2.06	0.106
		E X T	0.37	0. 861
	*P. umbilicalis*	Emersion	1.65	0.211
		Time of Day	4.80	**<0.01**
		E X T	5.70	**0.001**

3 Control vs. 90% water loss.

Significant differences are shown in bold with p values.

#### Nitrate uptake and nitrate reductase (NR) activity

**Figure 6:**
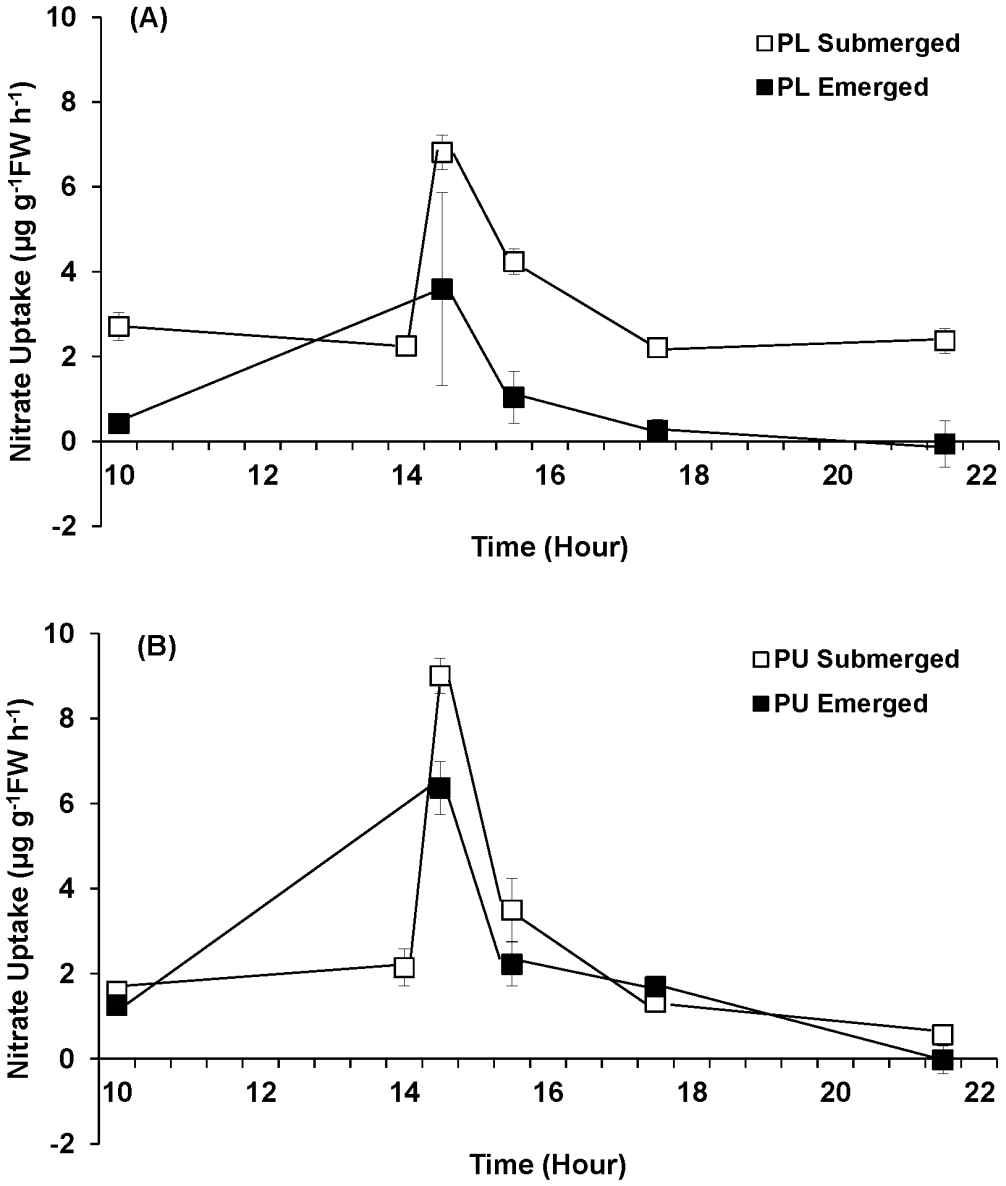
Nitrate uptake of *Porphyra linearis* (PL) (A) and *P. umbilicalis* (PU) (B) from the upper intertidal zone during nitrate reductase experiments using thalli collected from the early winter (Nov. -Dec.) population. *Porphyra* was cultivated under ambient sunlight in a greenhouse, with sunrise and sunset at ca. 07∶00 and 18∶00, respectively. Filled squares represent an emersion treatment which was exposed to air at a semi-diurnal cycle (10∶00–14∶00). All controls, open squares, remained submerged. Error bars represent ± one standard deviation.

**Figure 7:**
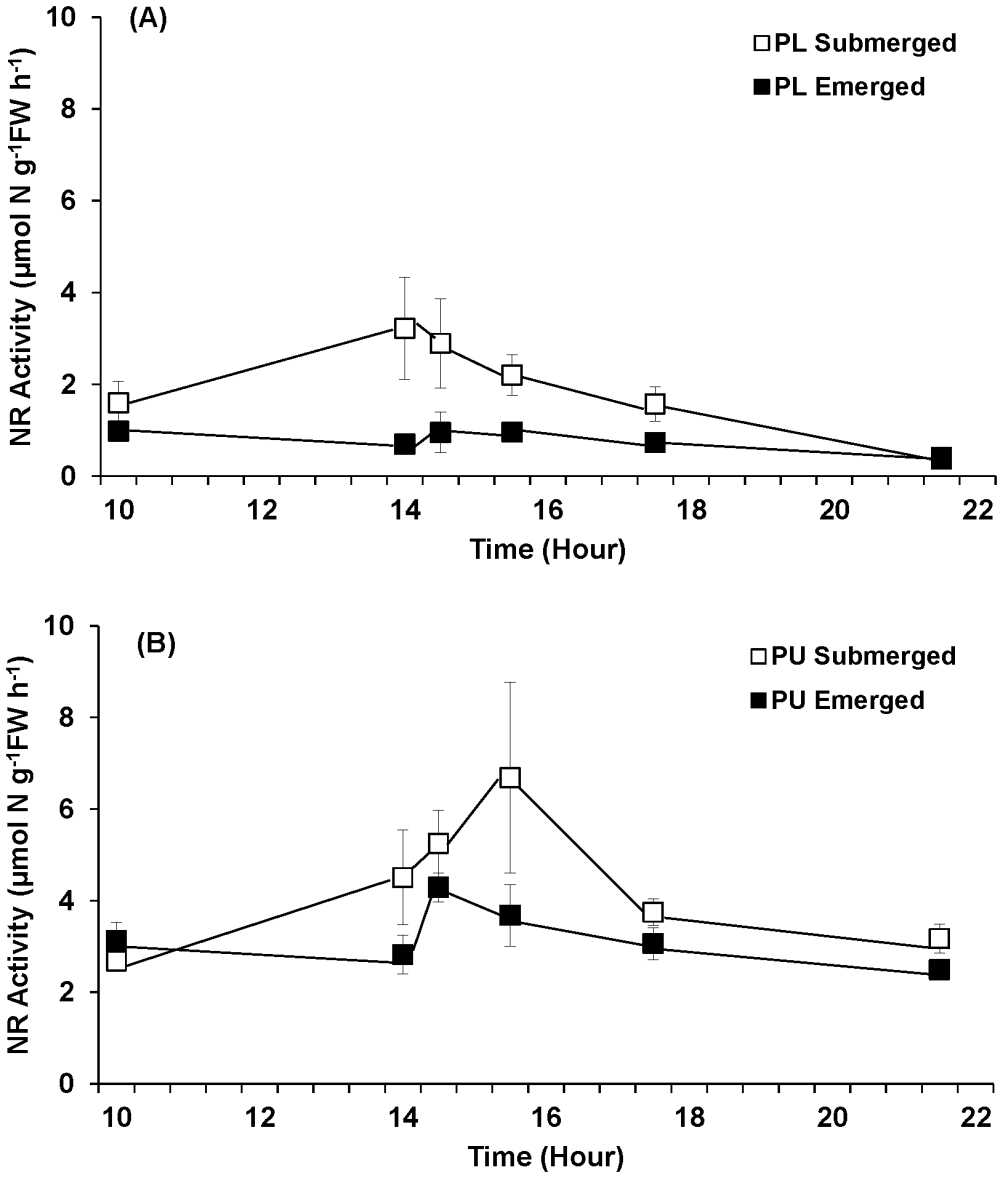
Nitrate reductase activity of *Porphyra linearis* (PL) (A) and *P. umbilicalis* (PU) (B) from the upper intertidal zone during nitrate reductase experiments using thalli collected from the early winter (Nov. -Dec.) population. *Porphyra* was cultivated under ambient sunlight in a greenhouse, with sunrise and sunset at ca. 07∶00 and 18∶00, respectively. Filled squares represent an emersion treatment which was exposed to air at a semi-diurnal cycle (10∶00–14∶00). All controls, open squares, remained submerged. Error bars represent ± one standard deviation.

Emersion significantly influenced both nitrate uptake and NR activity of *Porphyra linearis* and *P. umbilicalis* (Fig. 6, 7; Table 2). When the tissues were continuously submerged, the nitrate uptake and NR activity showed diurnal patterns with peaks at approximately 7.5–9 h after the start of lighted period. *Porphyra linearis* experiencing emersion exhibited nitrate uptake rates that were significantly (71%) lower than those experiencing continuous submergence. Time also affected NO_3_^−^ uptake by *P. linearis* the rate at 1030 h higher than all other time points, and 1530 h greater than 1730 h. The significant interaction between time and treatment for *P. umbilicalis* NO_3_^−^ uptake derived from the different response during the 30–90 min period post-emersion (Fig. 6) when the control (submerged) samples exhibited significantly higher rates of NO_3_^−^ uptake than the emerged samples (with no difference between submerged and emerged samples at the other time points). Overall, NO_3_^−^ uptake by *P. umbilicalis* from the emergent treatment averaged 59% lower than uptake under the control (submerged) conditions. On average, NO_3_^−^ uptake rates of *P. umbilicalis* were 16% greater than rates of *P. linearis*.

Treatment and time interacted to influence NR activity of *P. linearis;* uptake was significantly lower in emerged samples than submerged the end of emergence (1400 h) and at 30 and 90 min afterward (Fig. 7). Overall, NR activities of *P. linearis* and *P. umbilicalis* were within 5% of each other.

**Figure 8:**
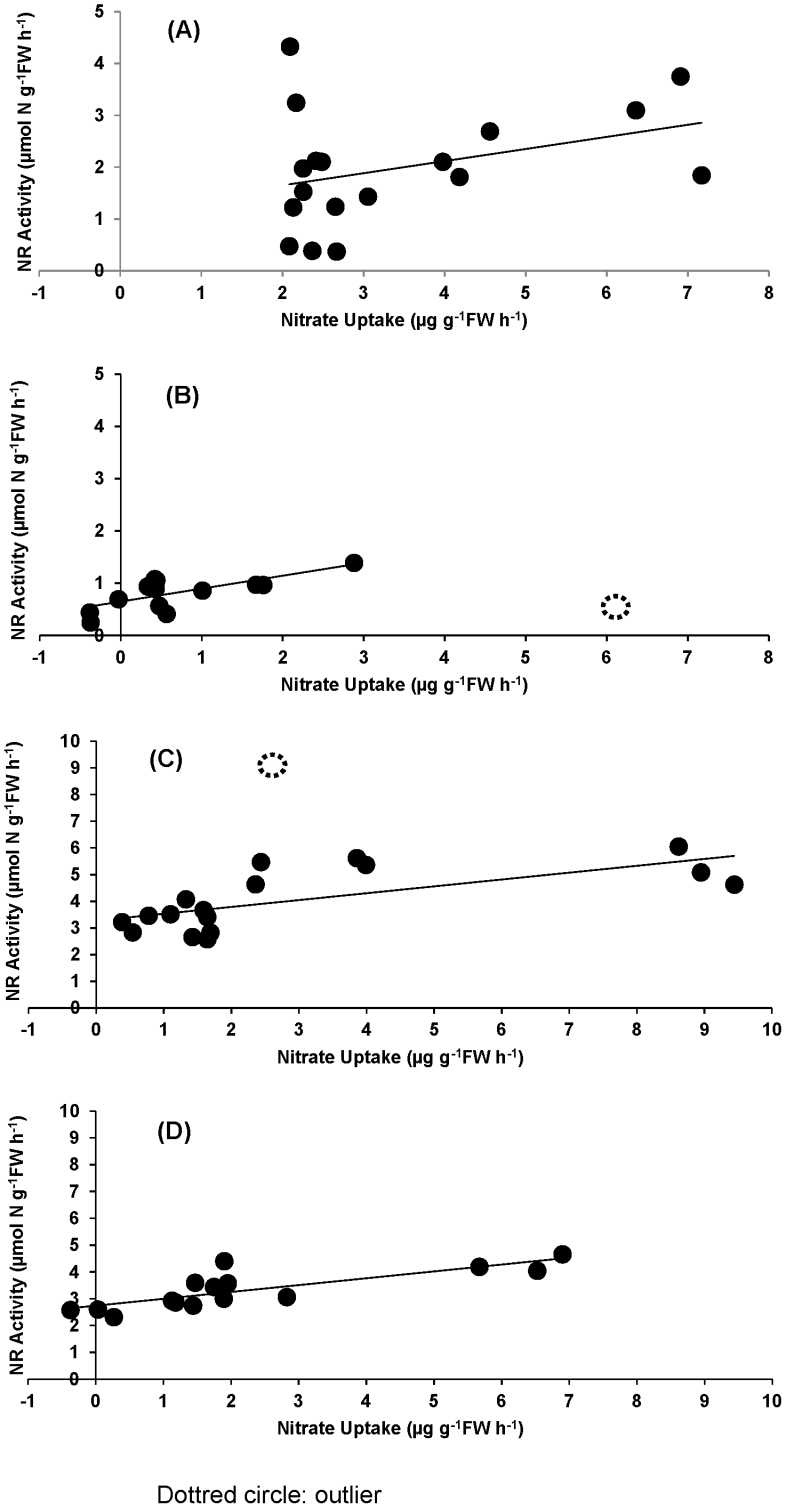
Nitrate uptake vs. NR activity of *Porphyra linearis* control (F_1,15_ = 6.72, p = 0.02 without outlier) (A), *P. linearis* emerged (F_1,12_ = 12.7, p = 0.003 without outlier) (B), *P. umbilicalis* control (F_1,15_ = 13.6, p = 0.002 without outlier) (C) and *P. umbilicalis* emerged (F_1,15_ = 21.2, p<0.0001) (D). Dotted circles represent the outliers.

Nitrate uptake and NR activity were significantly related in full pooled data sets only in emerged *P. umbilicalis* (F_1,12_ = 21.2, p<0.001; Fig. 8). However, when outliers were removed, NR activity was significantly and linearly related to nitrate uptake rate in submerged *P. umbilicalis* (F_1,15_ = 13.6, p = 0.0022) *P. linearis* under emersion treatment (F_1,12_ = 12.7, p = 0.0039). The slopes of the control (submerged) and experimental (emerged) treatments did not differ significantly (p>0.50) for *P. umbilicalis*.

### GS Experiments

#### Growth

**Figure 9:**
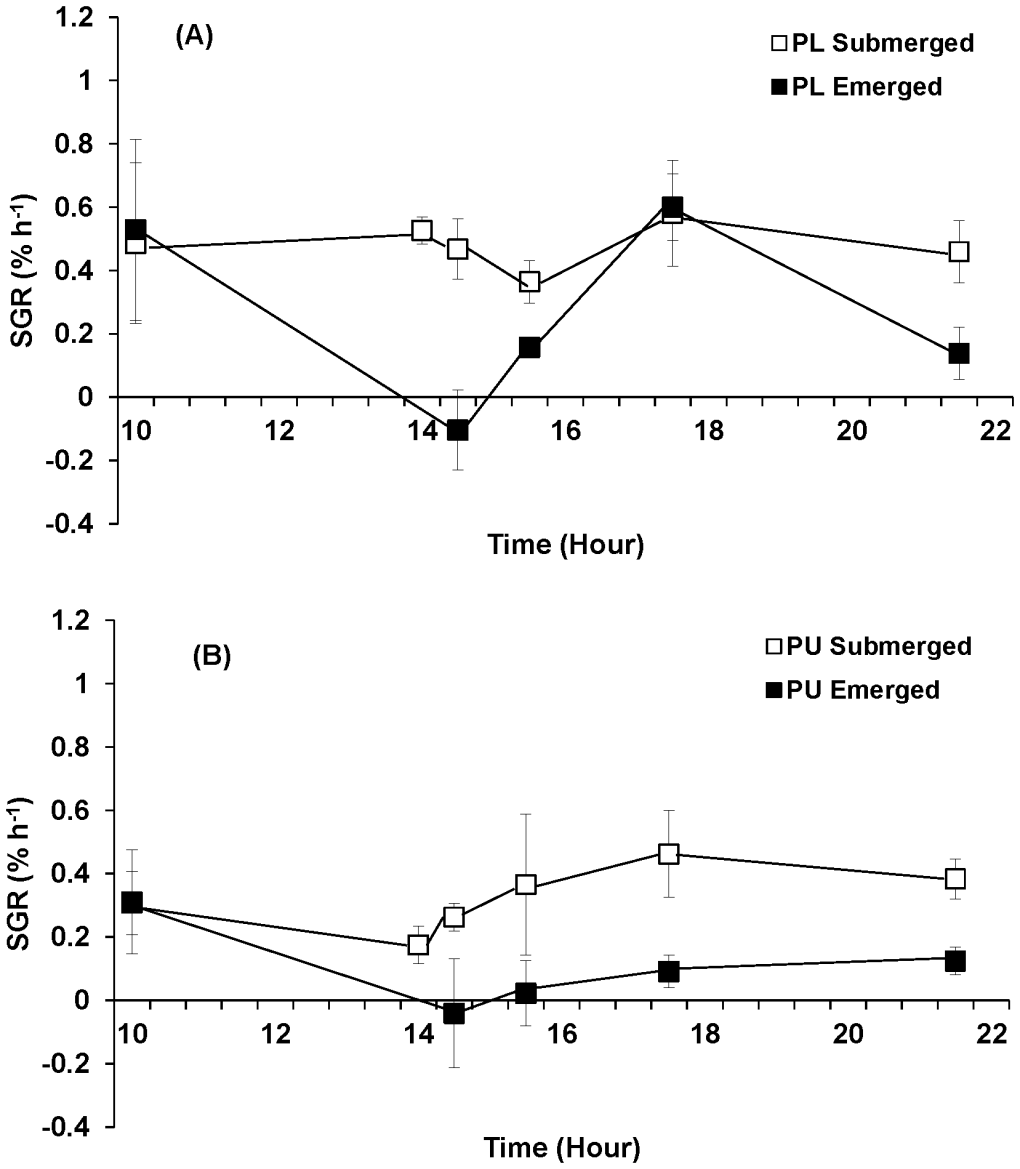
Growth rates of *Porphyra linearis* (PL) (A) and *P. umbilicalis* (PU) (B) from the upper intertidal zone during nitrate reductase experiments using thalli collected from the early winter (Nov. -Dec.) population. *Porphyra* was cultivated under ambient sunlight in a greenhouse, with sunrise and sunset at ca. 07∶00 and 18∶00, respectively. Filled squares represent an emersion treatment which was exposed to air at a semi-diurnal cycle (10∶00–14∶00). All controls, open squares, remained submerged. Error bars represent ± one standard deviation.

Growth rate at each sampling was again calculated against the initial weight at 0700 h. Emersion significantly affected the growth of both species (Table 2; p = 0.001 for *Porphyra linearis* and p<0.001 for *P. umbilicalis*; Fig. 9). When *Porphyra* tissues were continuously submerged, *P. linearis* grew 0.48% h^−1^ while *P. umbilicalis* grew on average 0.33% h^−1^. However, when *P. linearis* experienced 90% water loss during emersion, the growth rate was approximately 0.27% h^−1^ which is 55% of control rates, while *P. umbilicalis* grew 0.10% h^−1^, only 31% of control rates (Fig. 9).

#### Nitrate uptake and glutamine synthetase (GS) activity

**Figure 10:**
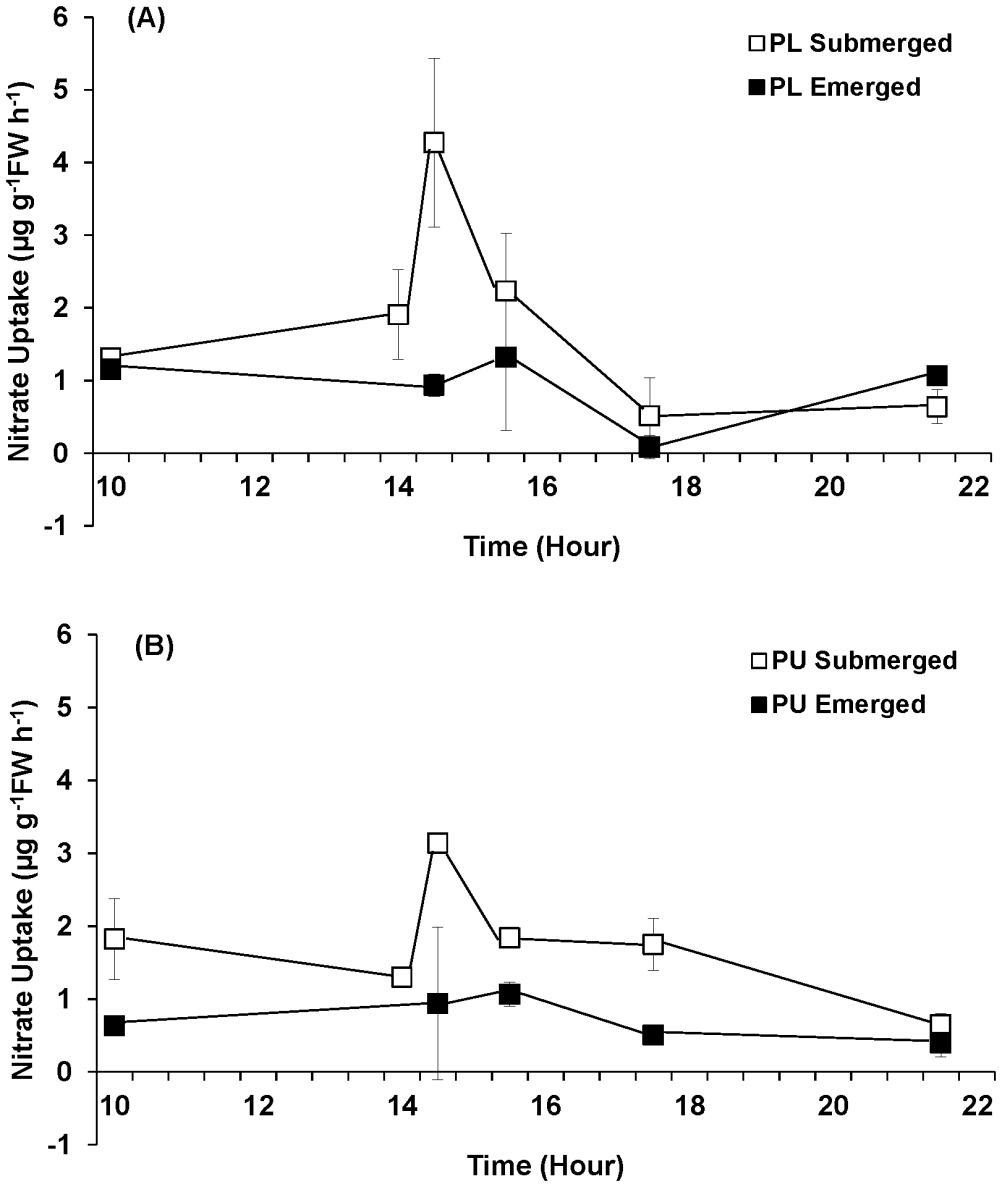
Nitrate uptake of *Porphyra linearis* (PL) (A) and *P. umbilicalis* (PU) (B) from the upper intertidal zone during nitrate reductase experiments using thalli collected from the early winter (Nov. -Dec.) population. *Porphyra* was cultivated under ambient sunlight in a greenhouse, with sunrise and sunset at ca. 07∶00 and 18∶00, respectively. Filled squares represent an emersion treatment which was exposed to air at a semi-diurnal cycle (10∶00–14∶00). All controls, open squares, remained submerged. Error bars represent ± one standard deviation.

Consistent with the parallel NR experiments, emersion significantly influenced NO_3_^−^ uptake in both *Porphyra* species during the GS experiments; both *P. linearis* and *P. umbilicalis* showed peaks in NO_3_^−^ uptake in the middle of lighted period, followed by a decrease in the uptake rate (Fig. 10; Table 2). Time and treatment interacted significantly to influence the rate of NO_3_^−^ uptake by *P. linearis.* The significant interaction between time and treatment resulted from the mid-cycle (1430 h) elevation in uptake rate by submerged samples not seen in the emerged samples (treatment did not affect NO_3_^−^ uptake by *P. linearis* at the other time points). Likewise, time and treatment interacted significantly to influence the NO_3_^−^ uptake by *P. umbilicalis.* However, the interaction effect derived from the difference between submerged and emerged treatments across the experiment (1000, 1430, and 1730 h; Fig. 10).

**Figure 11:**
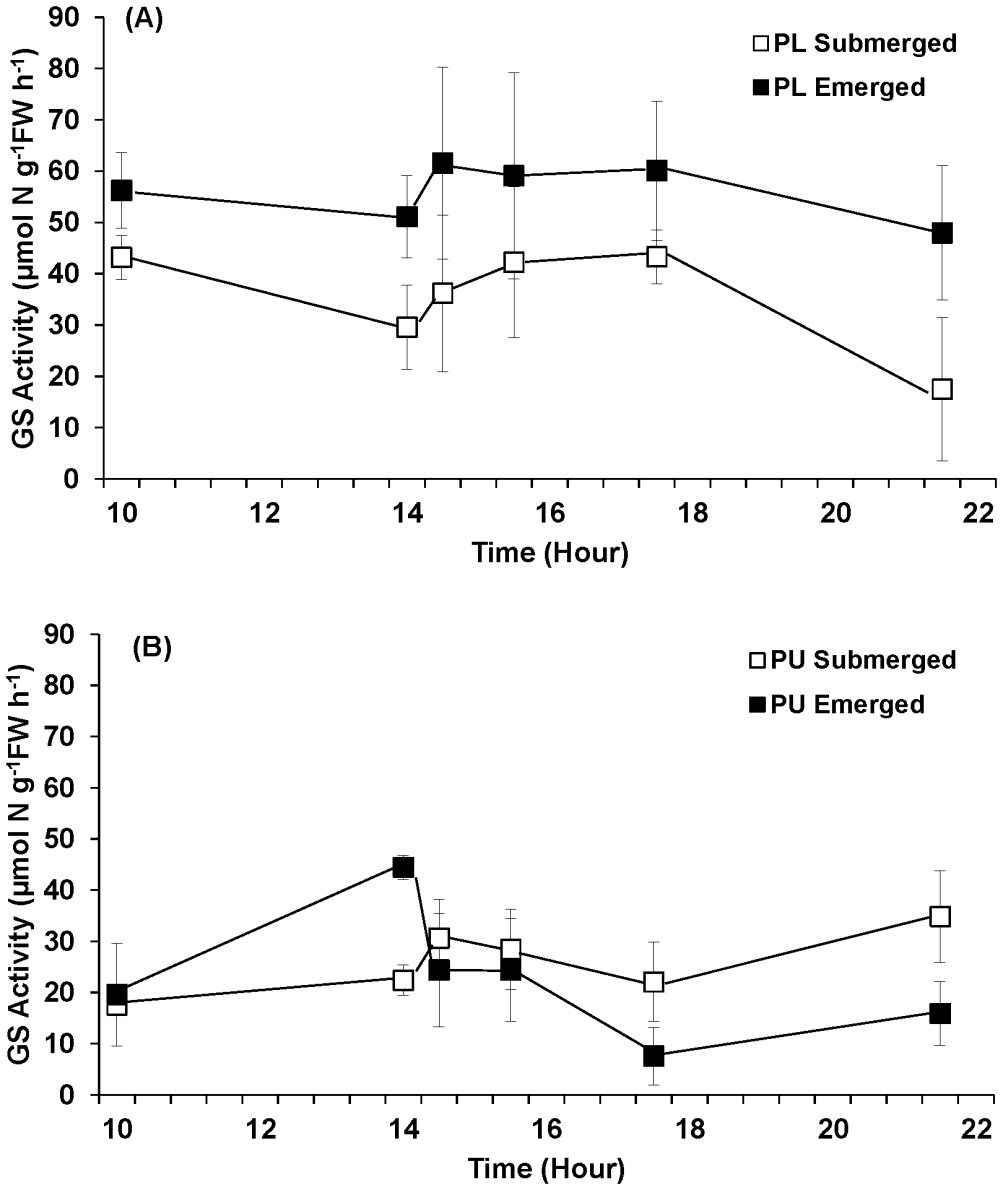
Glutamine synthetase activity of *Porphyra linearis* (PL) (A) and *P. umbilicalis* (PU) (B) from the upper intertidal zone during nitrate reductase experiments using thalli collected from the early winter (Nov. -Dec.) population. *Porphyra* was cultivated under ambient sunlight in a greenhouse, with sunrise and sunset at ca. 07∶00 and 18∶00, respectively. Filled squares represent an emersion treatment which was exposed to air at a semi-diurnal cycle (10∶00–14∶00). All controls, open squares, remained submerged. Error bars represent ± one standard deviation.

Treatment significantly affected the GS activity of *P. linearis* (Fig. 11, Table 2), with activities in the emergent samples averaging 59% higher than those under the continuously submerged treatment. GS activity of *P. linearis* did not vary significantly across the experiment. The GS activity of *P. umbilicalis* was significantly influenced by time during the experiment; activities averaged 80% higher in emerged samples at the end of the period of emersion (1400 h), compared with control (submerged) samples, and 20% lower than controls samples 3.5 h after the emersion period. GS activity was not correlated with either NO_3_^−^ uptake or NR activity in either *Porphyra* species (data not shown).

## Discussion

Kim et al. [2] reported that exposure to air during simulated tidal emersion induced a reduction in tissue N in three *Porphyra* species. Our follow-up study confirms that report for *P. umbilicalis* and extends our understanding of one possible mechanism. The decline of tissue N over a 4 h period of emersion was 12.0%, 9.1%, and 22.2% for *P. umbilicalis, P. leucosticte* (currently regarded as a taxonomic synonym of *Pyropia leucosticta*), *P. yezoensis* ( = *Pyropia yezoensis*), respectively [2], with the decrease for *P. umbilicalis* quite close to that for the current study (15.9%). The generality of the *Porphyra* tissue N loss during emersion argues for further study; the biogeochemical significance of N loss from these and other intertidal seaweeds is potentially quite large. Assuming a conservative biomass density of 1 mg DW cm^−2^ [37], the measured emersion-induced loss of N scales to a loss of ca. 0.021 g N m^−2^ h^−1^. If the vertical distribution of *P. umbilicalis* encompasses 5 m of the intertidal zone surface, this species alone could return to the environment 0.10 g N h^−1^ for each meter along the coastline.

The form of N returned to the environment (gaseous N_2_, nitrate or ammonium, or organic N) is important to determining what compartments of the biogeochemical cycle of N are most influenced by this loss from intertidal seaweeds. To investigate one mechanism of N loss, we measured δ^15^N before, during and after emersion. Law et al. [9] reported that denitrification occurred by epiphyton on the surface of the macroalgae *Ulva* (formally called ‘*Enteromorpha’*) and *Fucus* in the Tamar estuary, SW England. Denitrifying bacteria reduce ^14^N preferentially over ^15^N [14]. Therefore, if bacteria in association with *P. umbilicalis* were denitrifying NO_3_^−^, we would expect an increase in isotope fractionation in tissue during emersion. Our results revealed no discrimination against ^14^N during the 4 h period of emersion. However, acclimation to the treatment regimes prior to experimentation produced different isotopic signatures (continuously submerged vs. tidally emerged), visible at the start of the experiment (Fig. 4).

In addition, water loss by the algal thallus (here up to 95%) subjected the associated microbiota to similar desiccation stress. In general, desiccation of prokaryotes rapidly inhibits metabolism [38], [39] and is often lethal [40]. We conclude that N release from *Porphyra* during emersion does not occur via bacterial denitrification.

Though on balance the evidence argues against denitrification from the *P. umbilicalis* thallus as the loss mechanism, one possible alternate involves photorespiration. Zou and Gao [41] reported that the CO_2_ compensation point of *Porphyra* increased during emersion due to the enhanced photorespiration. The photorespiration pathway is tightly linked to N assimilation. During photorespiration, proteins in mitochondria are deaminated and the photorespiratory NH_4_^+^ is transferred to chloroplasts where it is re-assimilated by glutamine synthetase (GS) [42]. In higher plants, the reassimilatory flux of NH_4_^+^ during photorespiration may be 10-fold greater than primary N assimilation [43], [44]. This N release by agricultural crops was estimated in the range of 0–4.1 kg N ha^−1^ y^−1^ [45]. Pearson et al. [44] also reported that the N release by that of a wild plant, *Mercurialis perennis*, could be 0.25–0.33 kg ha^−1^ y^−1^.

Re-assimilation rate should be closely related to GS activity. In barley, the re-assimilation of photorepiratory ammonium significantly increased as GS activity in leaves increased [46]. In the present study, we found that GS activity in *Porphyra umbilicalis* was higher in emerged individuals than in non-emerged individuals. This higher GS activity in emerged *P. umbilicalis* may represent an increased capacity to re-assimilate NH_4_^+^ produced during photorespiration. The decrease in tissue N and protein concentration during emersion suggests that N in an organic form catabolized and lost, at least in part via photorespiration. We suggest that a portion of the photorespiratory NH_4_^+^ is re-assimilated by *P. umbilicalis* in chloroplasts (evidenced by the elevated GS activity), but the balance is lost from the thallus as NH_3_, maybe through disrupted membrane by emersion [20]. This loss of N is supported by the difference in δ^15^N values between the control and emersion treatments at the start of the N loss experiment (Fig. 4). Tissues acclimated to the emersion treatment have a history of losing NH_3_ via volatilization during periods of emersion. The NH_3_ leaving the *Porphyra* thallus will be enriched in the lighter N isotope (^14^N), in a manner analogous to that of the fractionation of ^16^O and ^18^O during evaporative losses of water [47]. This leaves the remaining tissue N enriched in ^15^N relative to the continuously submerged control.

Using data on GS activity and the change in tissue N during emersion, we estimated the amount of NH_4_^+^ produced by photorespiration (re-assimilated NH_4_^+^+emitted NH_3_). To do so, we assumed that NH_4_^+^ produced by photorespiration during emersion was not derived from NO_3_^−^; no N uptake occurred during emersion and the amount of intracellular NO_3_^−^ is insignificant [48]. Therefore, photorespiration was the only source of NH_4_^+^ for glutamine synthesis, and all photorespiratory NH_4_^+^ was either re-assimilated by GS or released into the ambient environment. In our study, the average *in vitro* GS activity during 4 h of emersion was 32 µmol N g FW^−1^ h^−1^. On average 8.3 mg N was lost per gram FW of *P. umbilicalis* during 4 h of emersion. This translates into 1.66 mg of N in 1 g FW of *P. umbilicalis* was lost from the thallus (assuming DW:FW is 0.2), or approximately 25 µmol N released g^−1^ FW h^−1^ (MW of NH_3_ = 17.03). Therefore, the total photorespiratory NH_4_^+^ generated during emersion would be 57 µmol N g FW^−1^ h^−1^. Approximately 44% of photorespiratory NH_4_^+^ appears to be lost. Since GS activity measured in an *in vitro* assay may be several-fold greater than the *in vivo* rate of NH_4_^+^ assimilation [49], the estimate of the portion of NH_3_ lost is conservative (i.e., the actual amount of N lost may be greater). This high rate of NH_3_ release resulted in a loss of protein and a decline of tissue N in *P. umbilicalis*.

In the present study, we found not only a reduction in tissue N, but also a reduction in tissue protein content during emersion; tissue protein was significantly correlated with tissue N. With the evidence of the large fraction of N present in seaweed as protein (67.6%∼98.3% of total N; [48]), the results of the present study support our hypothesis that organic N-containing compounds in *P. umbilicalis* tissues are degraded, with loss of N to the environment during emersion. However, although emersion caused a reduction in N and protein levels, tissues that had experienced periodic (i.e., tidal) emersion had higher levels of tissue N [2, this study] and protein [this study] than thalli that had been continuously submerged. This elevated protein content could result from synthesis of emersion tolerance proteins (e.g. dehydrins) [50]. The emersion-induced loss of N and protein in tissue of *P. umbilicalis* was matched by the recovery of both indices over the following 16 h (including resubmergence, 4 h "nighttime" emersion, and resubmergence until 1000 h; Figs. 1, 2). Lacking samples during the intervening 16 h, we cannot know the kinetics of the recovery process. The homogeneity of the slopes of *P. umbilicalis* relating NO_3_^−^ uptake and NR activity under both submerged and emerged treatments (Fig. 8) suggest a widespread effect of emersion stress on N metabolism. The connection between NO_3_^−^ uptake and NR activity is well known in other plants [51], [52]. Clearly, though, *P. umbilicalis* possesses the mechanisms to tolerate and recover from the stresses associated with emersion.

The findings from the present study expand our understanding of the global N cycle. Although intertidal seaweed communities have been long-studied, the potential to emit nitrogen into the atmosphere has not been recognized previously. If N release during emersion occurs in other intertidal and/or aquacultured species, the N contribution to the atmosphere by intertidal seaweeds could be significant if loss rates are similar to that of *Porphyra umbilicalis*. However, what is clear from the comparison of the responses of *P. umbilicalis* and *P. linearis* to emersion stress is the existence of significant interspecific differences in N metabolism and possibly loss. In the present study, the early winter populations of these two species with different thickness but from the same elevation, showed different physiological responses. When these two *Porphyra* species, collected in early winter (November-December), were cultivated under the continuously submerged condition, *P. linearis* and *P. umbilicalis* showed growth rates of 0.84% and 0.48% h^−1^, respectively, similar to those reported in the previous studies [26], [28], [29]. When exposed to emersion stress, *P. linearis* grew only 0.08% d^−1^, approximately 10% of the control value, while the growth rate of *P. umbilicalis* was 55% of control. The mid-winter populations (January-February) of both species, however, showed similar responses of growth, nitrate uptake and GS activity to the emersion stresses. This suggests seasonal, population level variability in physiological tolerance of emersion stress [53]–[55]. The difference in tolerance may reflect different environmental histories [20]. *Porphyra linearis* grows from late fall to spring, and by early summer, the thalli of this alga disappear, while *P. umbilicalis* occurs throughout the year [26], [56]. This signifies that the early winter population of *P. linearis* was comprised of newly formed thalli, with little experience of emersion stress, while *P. umbilicalis* population experienced severe summer/fall emersion stress in the upper intertidal zone.

We have confirmed N loss from intertidal *P. umbilicalis* during emersion. The N lost from *Porphyra* appears to derive, at least in part, from the catabolism of protein. Nitrogen release from *P. umbilicalis* during emersion does not occur via denitrification, but may be via photorespiration. This is the first study showing a possibility of N (e.g. NH_3_) release into the atmosphere by an intertidal seaweed. We also found differing physiological responses to emersion stress in different *Porphyra* species from the same intertidal elevation. This may depend on the conditions they have experienced in the past, rather than the morphological features (e.g. surface area-volume ratio). To further evaluate N loss by intertidal seaweeds, the form of the post-emersion loss of N must be identified, and other seaweeds should be tested to assess the generality of the loss. Particular attention should be paid to seaweeds, like *Porphyra*, that are desiccated routinely during aquaculture operations in Asia. Using the rate of tissue N loss measured in a prior study [2], and the biomass production measured in He et al. [57], we estimate that *Pyropia yezoensis* may return to the environment more than 150 times as much N (640 kg N ha^−1^ y^−1^) as lost from terrestrial plants (4.1 kg N ha^−1^ y^−1^; [45]). Given the magnitude of these operations, seaweed aquaculture may include a hitherto unappreciated anthropogenic impact on the global N biogeochemical cycle.
